# Quaternary tufas of the western Potiguar Basin, Brazil: rapid xeromorphic adaptation and climate change inferred from sedimentology, paleobotany, and fossil diagenesis

**DOI:** 10.1007/s00114-026-02098-z

**Published:** 2026-05-25

**Authors:** Tito Aureliano, Lorenzo Correa, Aline M. Ghilardi, Marcelle Erthal, Tarsila B. Dantas, Cayo C. C. Pontes, Marciana L. Lima, Rubson Maia, Bruno Belila Rusinelli, Francisco Santiago, Francisco P. Lima-Filho, Fresia S. Ricardi-Branco, Francisco H. R. Bezerra

**Affiliations:** 1 Universidade Federal do Rio Grande do Norte, Natal, Brazil; 2 Campinas University, Campinas, Brazil; 3 Centro de Pesquisa da Petrobras, Rio de Janeiro, Brazil; 4 Universidade Federal do Ceará, Fortaleza, Brazil

**Keywords:** Potiguar Basin, Paleoclimate, Porosity, Microbialites, Petrography

## Abstract

Quaternary tufa carbonates from Brazil’s Potiguar Basin provide unique insights into the interplay of biogenic, hydrological, and climatic factors driving continental carbonate precipitation in tropical low-energy environments. This study systematically characterizes the depositional, taphonomic, and diagenetic features of the tufa deposits resulting from the dissolution of rocks from the Formação Jandaíra that overlie the lithotypes of the Formação Açu at the Quixeré locality (westernmost Potiguar Basin). We employed a multi-scalar analysis encompassing macroscopic description, petrographic thin-section analysis, scanning electron microscopy (SEM), paleobotanical analysis, and digital porosimetry. Paleobotanical analysis of leaf morphotypes quantifies a major climatic transition, from a humid tropical paleoenvironment that supported a mesophytic forest (MAT: 25.4–25.9 °C; MAP: 515–779 mm/year) to the modern xeromorphic Caatinga biome. This study indicates that the Quixeré tufas are high-resolution archives that concurrently record the sedimentological response to fluctuating depositional energy, the diagenetic pathways of continental carbonates, and the ecological turnover driven by late Quaternary aridification. The findings provide a model for interpreting tropical paleoenvironments and understanding the development of complex pore systems in heterogeneous carbonate successions.

## Introduction

Today, the Caatinga biome stands as South America’s largest seasonally dry tropical forest, renowned for its extreme aridity and ecological vulnerability in a vast semiarid area that spans northeastern Brazil (Albuquerque et al. 2012; M. G. Santos et al. 2014). This region experiences unpredictable rainfall, extended droughts, and escalating temperatures, trends that are forecasted to intensify due to climate change and ongoing land use pressures (Fernandes et al. 2022). The modern Caatinga is characterized by xeromorphic vegetation displaying remarkable physiological and anatomical adaptations, enabling survival in nutrient-poor soils with persistent water deficits (Araujo et al. 2025; Moura et al. 2025). Leaf and wood traits such as reduced surface area, high sclerophylly, and water-conserving morphologies reflect a striking evolutionary response to the region’s chronically dry climate (Dória et al. 2016). Palynological evidence and modern ecological surveys indicate that these adaptations have become progressively more pronounced since the Late Pleistocene and early Holocene, as semiarid conditions have advanced throughout northeastern South America (Fernandes et al. 2022). However, fossil-rich sedimentary outcrops across northeastern Brazil, including those in the Potiguar Basin, preserve a contrasting image of the past climate regime. Within Quaternary tufa deposits, fossil leaf morphotypes and stem remains are often found, exhibiting forms and sizes consistent with a more humid tropical environment than seen in the present-day Caatinga (Fernandes et al. 2022).

Tufa systems, which form natural pools rimmed with diverse plant assemblages and sometimes shaded by relict forested margins, serve as key natural laboratories for studying both paleohydrological and biogeochemical processes in continental carbonates (Ford and Pedley 1996; Pentecost and Viles 2007). Rapid precipitation of calcite in these settings encapsulates and preserves plant debris, resulting in highly porous karstic architectures and fossiliferous carbonate bodies. The early diagenesis of tufa deposits is characterized by synchronous lithification and intense chemical alteration of the carbonate cement, often accompanied by organic decay (Oste et al. 2023). This interaction results in the exceptional preservation of the leaf and stem molds, and upon subsequent removal or degradation, the development of large primary porosity in the rock (Barbosa 2013).

Riverine tufa deposits are recognized globally as critical archives of continental Quaternary climate change, providing high-resolution proxies for both hydrological and ecological shifts (Andrews 2006; Goudie, 2013). However, the majority of well-studied tufa systems are concentrated in temperate regions of Europe and North America, leaving a significant knowledge gap regarding their formation, diagenesis, and paleoenvironmental significance in tropical semi-arid settings (Ford and Pedley 1996; Pedley et al. 2003). These semi-arid landscapes, which are expanding globally, represent environments where the initiation and cessation of tufa growth are acutely sensitive to hydrological thresholds controlled by climate variability (Goudie, 2013). Understanding the sedimentological and diagenetic evolution of tufas in these water-stressed regions is therefore crucial for refining global paleoclimatic models and for predicting how fluvial and carbonate systems will respond to future aridification. The Quaternary tufas of Northeast Brazil, situated in one of the world’s largest and most ecologically sensitive semi-arid zones (Fernandes et al. 2022), thus offer a unique and vital opportunity to develop a depositional model that can be applied to other ancient and modern semi-desert regions worldwide, addressing a fundamental question in carbonate sedimentology. The present study examines a fossil-rich tufa occurrence in Quixeré, State of Ceará, providing comparative descriptions of preserved facies, fossil flora, and petrographic textures. By integrating sedimentology, paleobotany, and petrographic analyses, this study aims to clarify the diagenetic pathways, draw distinctions between modern semi-arid and ancient humid systems, and chart how shifts in microfacies and vegetational morphotypes reflect larger paleoclimatic and environmental dynamics throughout the Quaternary in the Brazilian semiarid region.

## Geological setting

### Regional geology

The Potiguar Basin represents a significant Mesozoic-Cenozoic sedimentary province located on the equatorial margin of Brazil, occupying a total area that includes both extensive subaerial and submerged portions (Pessoa-Neto et al. 2007). The studied region is geologically remarkable due to the occurrence of the Cretaceous Jandaira Formation, a carbonate-dominated succession within the post-rift supersequence, which records transitions from shallow marine to subaerial and marginal facies (Pessoa-Neto et al. 2007; Reyes-Pérez 2003). The Jandaira Formation exhibits characteristic limestones and dolostone, often containing evidence of Quaternary early diagenesis (e.g., dolomitization, karstification), major tectonic reactivations, and long-lasting exposure surfaces—all key elements favoring continental tufa precipitation (Arenas-Abad et al. 2010; Pessoa-Neto et al. 2007).

Episodes of faulting and uplift have repeatedly affected the stratigraphic architecture, favoring the circulation of meteoric water through fracture zones and the generation of karstic networks. As a result, collapsed paleocaves, tectonic valleys, and prominent structural depressions developed, controlling both the geometry and hydrology of the basin from the Cretaceous to recent times (Maia and Bezerra 2014; Mousa-Lima 2010). This tectono-sedimentary context has directly governed the deposition of Quaternary tufa deposits and associated paleolake, spring, or palustrine carbonates across the region (Maia and Bezerra 2014).

Palynological studies conducted in underlying and lateral units revealed the region’s climatic evolution from a hot, semi-arid habitat during the Cretaceous (Dino 1992). The overlying Cenozoic unconsolidated covers mostly comprise alluvial and colluvial sediments, as well as Quaternary tufas, depositing preferentially within structurally-controlled topographic lows (Maia and Bezerra 2014).

### Local geology

The Quixeré area, situated in the western Potiguar Basin, is typified by strong fracturing, intense neotectonic faulting, and the exposure of the Jandaira carbonate platform. The Jandaira itself, belonging to the Apodi Group, is laterally continuous and variable in thickness, with exposures characterized predominantly by micritic limestones and dolomitic strata interspersed with evidence of emersion (Pessoa-Neto et al. 2007; Reyes-Pérez et al. 2003). The region’s landscape is defined by collapsed karstic features and significant structural domains that collect and focus surface and subsurface drainage, creating ideal circumstances for precipitation of tufa carbonates (Diniz et al. 2022; Shiraishi et al. 2022).

The tufa bodies at Quixeré are situated on the western edge of the escarpment of the Potiguar Basin, and were preliminarily interpreted as deposits formed in waterfall and lacustrine-depression environments, subjected to intense fracturing and karstification, with the development of collapsed paleocave systems. These tufas are in direct contact with paleosols and siliciclastic rocks of the Açu Formation (Jesus 2012; Oste et al. 2021; Reis et al. 2014, 2015).

Petrographic investigation suggests significant early cementation, coupled with secondary dissolution and subsequent meteoric diagenesis. The topographic context and structural controls, in concert with the present semi-arid to sub-humid tropical climate, have promoted periodic but intense groundwater flows through fracture zones, further enhancing carbonate supersaturation and tufa precipitation (Payandi-Rolland et al. 2019; Shiraishi et al. 2022).

Subsurface geological mapping identifies the Jandaira Formation as a critical aquifer horizon and a potential reservoir analog thanks to its high primary and secondary porosity, developed during both deposition and multiple diagenetic phases (Pessoa-Neto et al. 2007). Importantly, local stratigraphy also includes the underlying Alagamar and Açu formations, which, although not directly outcropping in Quixeré, influence the local hydrological compartmentalization and control meteoric water movement (Dino 1992; Pessoa-Neto et al. 2007).

**Fig. 1:**
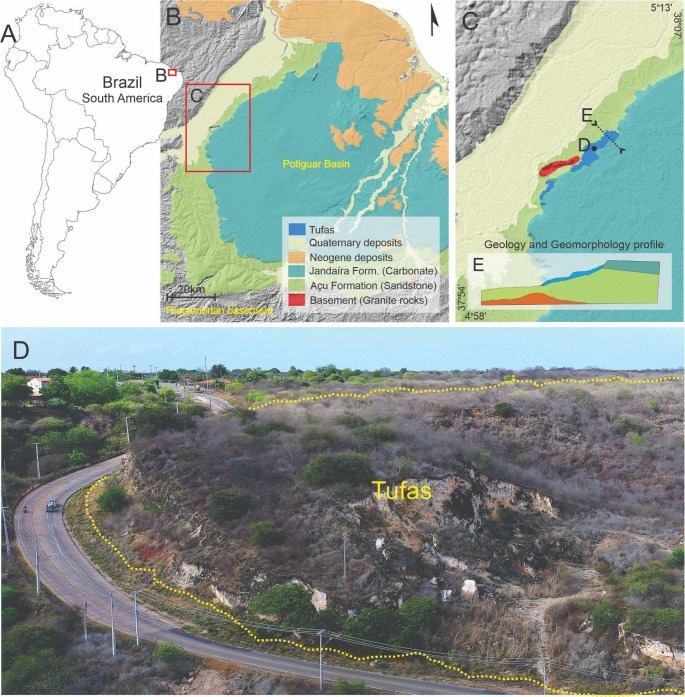
Locality of the Quaternary tufa deposits of Quixeré in the context of South America, and in the western Potiguar Basin (**A**). **B**, panoramic view of the surrounding Caatinga savanna. **C**, aerial drone photography. **D**, schematic drawing showing the tufa layer

The Quixeré tufa deposits gained significance as a potential outcrop analogue for fractured carbonate reservoirs, featuring collapsed paleocave systems and depositional structures, such as phytoclast-rich horizons, that enhance porosity, along with crystalline crust features acting as potential flow barriers. These deposits thus provide a valuable window into the geometry, connectivity, and volumetrics of carbonate reservoirs in exposed settings, supplying parameters applicable to hydrocarbon exploration, aquifer characterisation, or CO₂ storage contexts (Fig. 1).

## Climate evolution in the quaternary of Brazil

The Quaternary in Brazil, particularly in the semiarid northeastern region, was marked by pronounced climatic fluctuations driven by orbital forcing, glacial-interglacial cycles, and shifts in atmospheric circulation patterns (Behling 2002; Cruz et al. 2005). During the late Pleistocene, paleoclimatic evidence from speleothems, lacustrine sediments, and fossil records indicates that much of northeastern Brazil experienced significantly wetter conditions than today, with mean annual precipitation values often exceeding those of the modern semiarid Caatinga biome (Behling 1998; Behling et al. 2004). The Last Glacial Maximum (LGM), spanning approximately 26,000 to 19,000 years before present, was characterized by substantial reorganization of the South American Monsoon System (SAMS), with the position and intensity of the Intertropical Convergence Zone (ITCZ) playing a critical role in regional precipitation distribution (Cruz et al. 2005; Wang et al. 2004). Speleothem records from central and northeastern Brazil reveal that periods of enhanced rainy seasons activity during the late Pleistocene resulted from increased austral summer insolation, which strengthened convective rainfall and promoted the expansion of more humid tropical forests into areas now dominated by xeromorphic vegetation (Cruz et al. 2005; Stríkis et al. 2011). Heinrich events and other millennial-scale climatic oscillations in the North Atlantic further modulated South American precipitation patterns, with cold episodes in the Northern Hemisphere typically enhancing SAMS activity and increasing rainfall over tropical Brazil (Auler et al. 2004; Wang et al. 2004).

The Pleistocene-Holocene transition (~ 11,700 years ago) marked a pivotal shift in the climatic regime of Northeast Brazil, with progressive aridification becoming the dominant trend throughout the Holocene (Behling 2002; Chiessi et al. 2021). Palynological and sedimentological evidence from multiple sites across the region documents a replacement of mesophytic vegetation communities by increasingly xeromorphic assemblages adapted to water stress and prolonged dry seasons (Behling 1995, 1997; Pennington et al. 2000). The early to mid-Holocene (approximately 11,000 to 5,000 years BP) is generally interpreted as an interval of climatic optimum in many parts of Brazil, with relatively high humidity supporting diverse forest vegetation, though some records suggest spatial heterogeneity in precipitation patterns across the continent (Behling et al. 2001; P. E. Oliveira et al. 1999). However, the mid-to-late Holocene witnessed a contraction of the ITCZ and weakening of the SAMS, leading to reduced rainfall over northern and northeastern Brazil (Chiessi et al. 2021). This aridification trend intensified during the late Holocene (last 4,000 years), establishing the modern semiarid climate that characterizes the Caatinga biome (Behling 2002; Ledru et al. 2006). The transition from humid tropical conditions to semiarid environments is recorded in tufa deposits, paleosols, and fossil assemblages throughout the region, with erosional unconformities often marking periods of intensified aridity and fluvial incision (Behling et al. 2004; Jeske-Pieruschka and Behling 2012).

The evolution of the Caatinga biome is thus inextricably linked to these Quaternary climatic oscillations, representing a geologically recent ecological response to progressive aridification (Pennington et al. 2000; Prado and Gibbs 1993). Fossil evidence demonstrates that the xeromorphic adaptations characteristic of modern Caatinga vegetation (e.g., reduced leaf area, high sclerophylly, and deciduous growth habits) became increasingly dominant only during the Holocene as precipitation declined and seasonality intensified (Santos et al. 2007). Ecological niche modeling and phylogeographic studies of Caatinga species indicate that the current distribution of seasonally dry tropical forests in Northeast Brazil reflects refugial dynamics during glacial-interglacial cycles, with populations expanding during drier glacial periods and contracting during wetter interglacials (Pennington et al. 2000; Prado and Gibbs 1993). The replacement of mesophytic forests by xeromorphic vegetation represents one of the most dramatic biome shifts in South America during the Quaternary, with implications for understanding both past biodiversity dynamics and future responses to anthropogenic climate change (Ab’Sáber 2003; Behling 2002). Projections based on climate models suggest that the semiarid region of northeastern Brazil will continue to experience increasing aridity, with potential expansion of desert-like conditions into areas currently classified as semiarid (Marengo et al. 2009), underscoring the vulnerability of this region to ongoing climatic trends that have been developing since the middle Holocene (Chiessi et al. 2021).

## Materials and methods

An integrated analytical strategy was adopted to assess the sedimentological, taphonomic, paleontological, and paleoclimatic variables present in the Quixeré tufa deposits. The experimental design incorporates classical field and laboratory techniques as well as advanced quantitative approaches, providing a robust multi-scalar analysis of original and diagenetic features.

A total of eight representative rock samples were collected from the major tufa bodies at the Quixeré abandoned quarry. Each sample was documented in situ using digital photography, with attention given to scale, orientation, and surface features. Standardized protocols for facies description were followed, with an emphasis on identifying domal, laminated, shrub-rich, and fossiliferous textural types. Diagnostic sedimentological criteria (e.g., grain size, bedding, bioclast packing, evidence for microbialite development) were systematically recorded. Samples were classified according to modifications of the Dunham carbonate classification, further incorporating tufa-specific lithofacies as described by Ford and Pedley (1996) and refined for continental carbonate systems by Arenas-Abad et al. (2010). Notable mold types (leaf, stem, gastropod), microbial textures, and porosity features were described both in situ and in collected samples, following the methods of Arenas-Abad et al. (2010), Oste et al. (2021, 2023), and Ford and Pedley (1996).

### Petrography

Petrographic thin sections were prepared from selected subsamples. We applied PaleoBOND^®^ penetrant stabilizer to improve resistance in the coated leaf sample. All samples were embedded in Araldite 2020^®^ epoxy resin. These sections include both non-laminated and blue-dye-impregnated polished slides to highlight both fabric and pore structures. Analyses were performed under transmitted and reflected light on an Axiovision A1 scope equipped with digital imaging (Institute of Geosciences, Campinas University), an Olympus BX53M, and a Meyer Instruments PathScan Enabler (Sismology Laboratory, Federal University of Rio Grande do Norte). Key microfacies elements were systematically described, including phytoclasts, peloids, crystal shrubs, cements, and microbial laminae, following Arenas-Abad et al. (2010). Special attention was paid to the type, distribution, and paragenesis of radial blocky and micritic cement generations. We also cataloged the occurrence, orientation, and preservation of bioclast molds, as well as inter- and intracrystalline pore types, following Oste et al. (2021). We also generated a flow diagram showing the interplay between the processes of lithification, fossil diagenesis, and karstification, all scripted and created in SankeyMATIC (Bogart 2014).

For cathodoluminescence analysis (CL), we used a Cathodyne CATHOD-SP02 beam from NetTec Scientific, operating at 15 kV and 200 µA in low vacuum (< 1,200 mBar), in a Leica microscope (DM2700M) with a ZOOM x2.5 and a NewTec IS3VIS-16 color camera. The cathodoluminescence images were processed with Cathodyne v2024.7.1.0 software. The CL analysis was performed at the Laboratory of Sismology (LabSis), the Federal University of Rio Grande do Norte.

### Scanning electron microscopy

Selected samples underwent further investigation via scanning electron microscopy (SEM) model JSM-IT500HR - JEOL from the Electron Microscopy Laboratory, IG, UNICAMP. The SEM operated in vacuum mode at an acceleration voltage of 20.0 kV and is equipped with an Energy Dispersive Spectroscopy (EDS) detector model EDX; EX-74600U4L2Q DRY SD30 - JEOL. These analyses clarified features below the optical resolution, including the microstructure of shrub crystals, the identification of distinct pore types, microbial filaments, and their association with mineralization, and distinctions between cementing phases (Fig. 4). The EDS also verified mineralogical composition (Bastianini et al. 2019; Reyes-Pérez et al. 2003).

### Paleobotanical and morphometric analysis

 A paleobotanical dataset was compiled by systematically documenting all fossil leaf impressions with over 50% of their lamina preserved (*n* = 36; Fig. 6; Table S1). Morphotypes were assessed according to Ellis et al. (2009), with classification based on size (microphyll, nanophyll, notophyll), margin (entire/dentate), venation pattern, and quantitative dimensioning (length, width, area). Primary and secondary venation, as well as taphonomic characters, were described for each morphotype (Ellis et al. 2009) 

Paleoclimatic variables were calculated using validated equations for mean annual temperature (MAT) via leaf margin analysis (LMA), and mean annual precipitation (MAP) via leaf area analysis (LAA), tailored explicitly for South American fossil floras (Hagen 2019; Santiago and Ricardi-Branco 2020; Toumoulin et al. 2020).

### Digital image analysis and porosity quantification

Porosity was measured using color-segmented analysis of high-resolution scanned images of thin sections. Each image was processed via histogram equalization in Python, enabling separation of cement, grain, and pore components according to precise RGB thresholding. The adopted routine classified pixels into categories (cement, pore, grain) and calculated total porosity as the ratio of pore pixels to total pixels, thereby providing a quantitative, reproducible measure of pore space across multiple fabric types (Vidal et al. 2019). All scripting for this analysis was implemented using Python 3.13.5 with NumPy (Harris et al. 2020), Scikit-learn (Pedregosa et al. 2011), and Matplotlib (Hunter 2007). All coding was performed in Spyder 6.0.7 (Raybaut 2025) and libraries were managed in Anaconda 2.6.6 (Anaconda, Inc., 2024).

All data used in the analyses are provided in the supporting information and have been deposited in the Morphobank repository and can be accessed through the link: https://www.morphobank.org/permalink/?P6161.

## Results

Over 10 thin sections were produced from the eight samples obtained from two sections of the tufa.

**Fig. 2:**
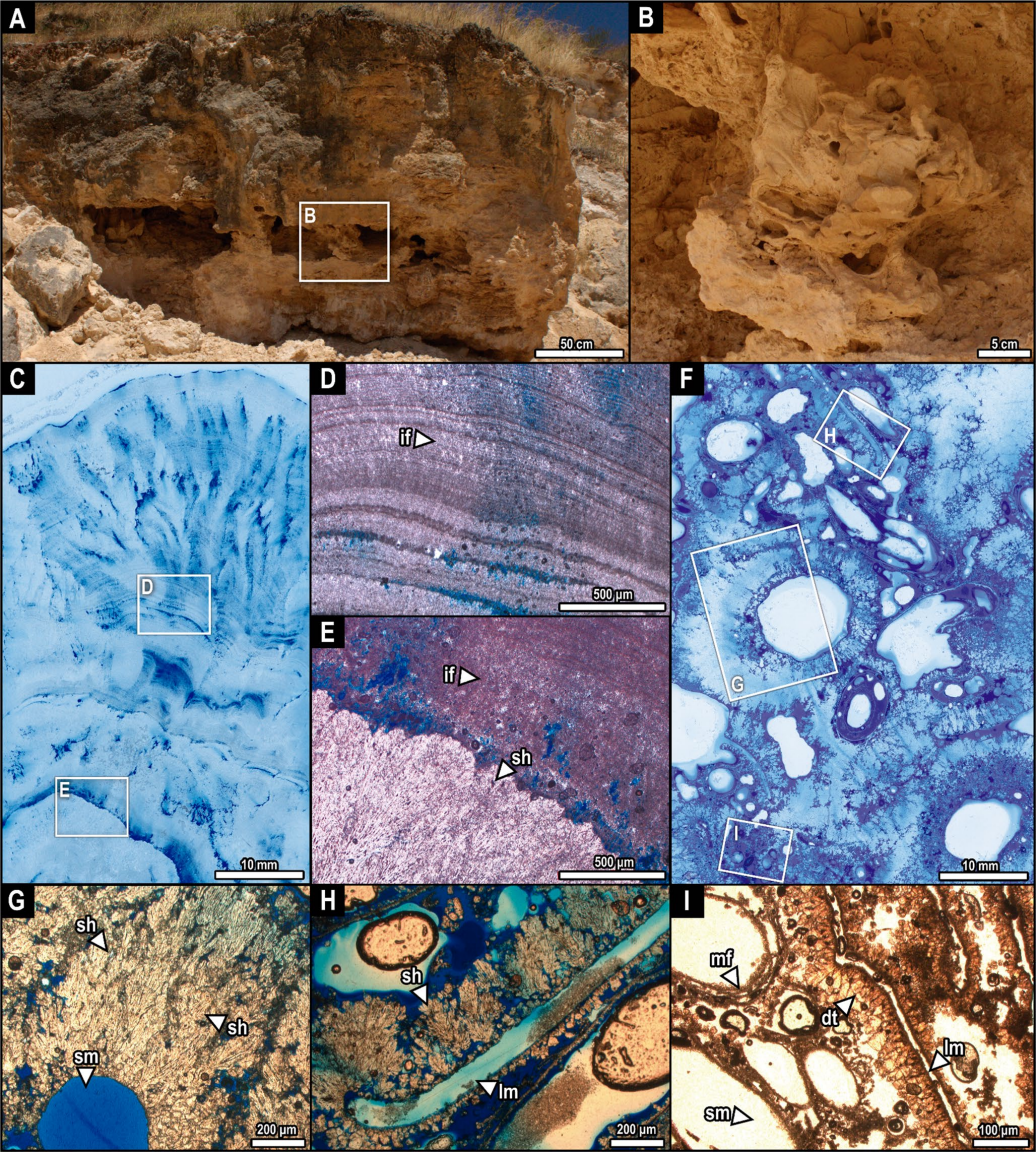
Outcrop of fossil tufa at the Quixeré site (outcrop 1; A) with detail of the phytoclastic rudstone bed (B). C, stromatolitic boundstone showing cyclic, biomediated, calcite spar (light tone) intercalated with micritic (dark) layers (E) locally, in the bottom of the stromatolitic facies (if), sparry shrub crust (sh) is observed. F, rudstone of phytoclasts, showing coated stems surrounded by radiating sparry shrubs (G), leaf mold surrounded by sparry shrubs (H), and stem molds covered by microbial filaments, and leaf mold with dog-tooth-like to bladed cloudy spar crystals (I). Note the microspar growing within biofilms around stem molds in I. Scans under normal light in C, F. Polarizing light under parallel nicols in D, E, G-I. **Abbreviations**: dt, dog-tooth-like to bladed spar calcite; if, isopachous fringe; lm, leaf mold; mf, microbial filament; sh, shrub; sm, stem mold

Outcrop 1 (Fig. 2) comprises a 2 m high outcrop that starts with 50 cm of interdigitated stromatolitic boundstones and phytoclastic rudstones. It is followed by 30 cm of a highly porous phytoclastic rudstone bed, characterized by lower and upper erosive contacts. There is notable karstification at this level, with extensive cavities ranging in width from 40 to 1000 mm. The topmost bed is a 100 cm repetition of the interdigitated stromatolitic boundstones and phytoclastic rudstones. The topmost bed comprises a pedogenic cover colonized by living vegetation.

**Fig. 3:**
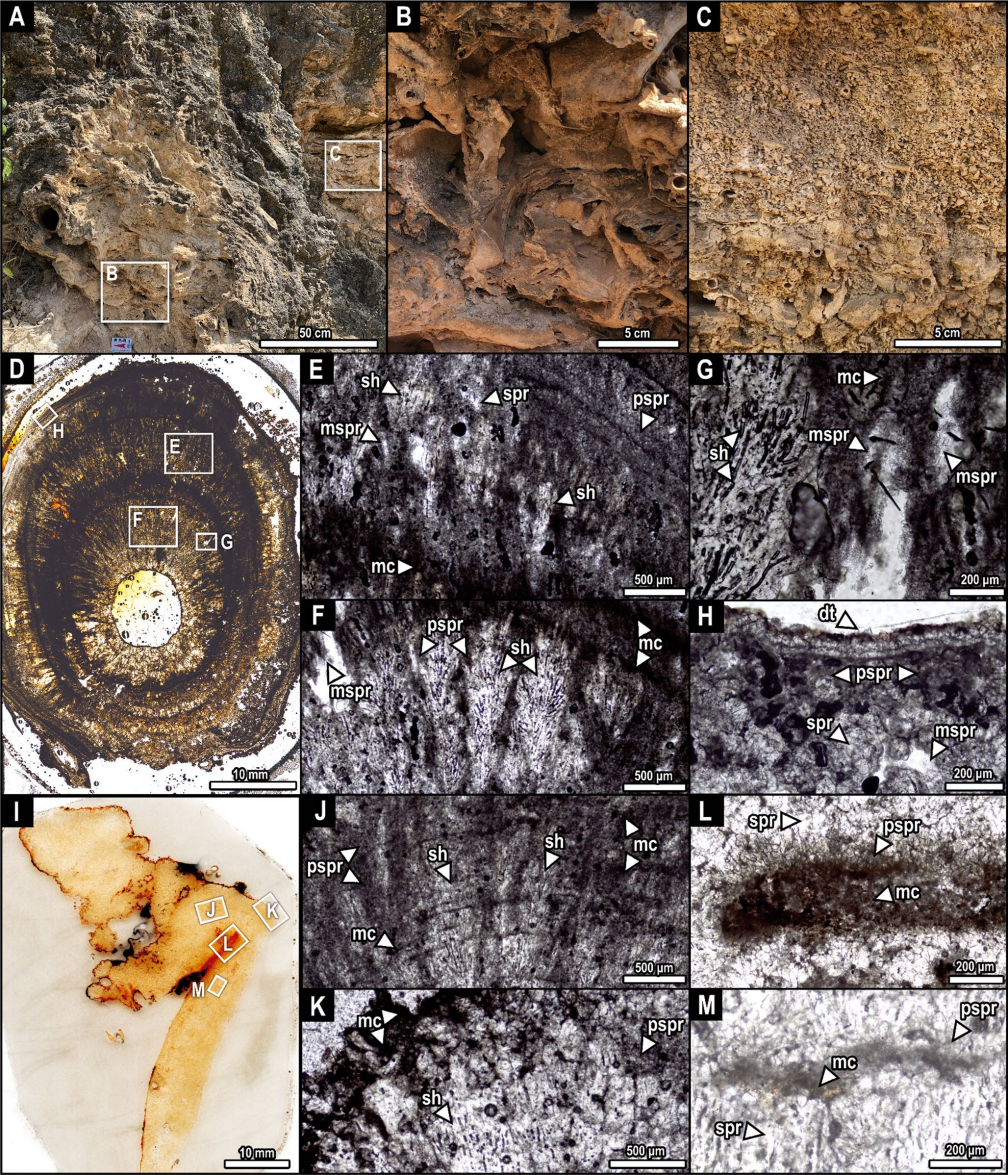
Outcrop of fossil tufa at the Quixeré site (outcrop 2; A) with detail of the phytoclastic rudstone beds (B, C). D, coated stem showing cyclic growths of intercalating shrub crusts and micrite (E-H). Detail of the shrub crusts and micrite, showing the co-occurrence of pseudospar and microsparite (E-H). Note the dogtooth aspect of the spar and microspar in H. I, leaf countercast showing the complex interplay of micrite, spar, microspar, pseudospar, and shrubs (J-M). The contact between spar and micrite is preserved in both graded (J, K) and non-graded (L, M) zones. Pseudosparite is more abundant in graded zones (J, K). Scans under normal light in D, I. Polarizing light under parallel nicols in E-H, J-M. **Abbreviations**: dt, dog-tooth-like to bladed spar calcite; mc, micrite; mspr, microsparite; pspr, pseudosparite; sh, shrub; spr, sparite

Outcrop 2 (Fig. 3; located 50 m from outcrop 1) is a 5 m-high succession of phytoclastic rudstones of varying macrophyte sizes, calcite-coated stems, distinct amounts of leaf countercasts, and varying amounts of spar cementation.

The surface presents signs of remobilization, similar to the ‘erosion’ or ‘remora’ type of ‘cascade’ (sensu Pentecost & Viles, 1994). However, a previous study reported the presence of collapsed paleocaves at this site (Reis et al. 2014), filled with macrophytes and other debris, providing a more accurate model for the present section. From the base to the top, there are large stems and chaotically arranged leaf countercasts (Fig. 3A, B). There is noticeable lateral discontinuity from 1.6 m to 2.0 m, where karstic cavities expose a level of phytoclastic rudstone with noticeably less cementation, smaller stem molds, fewer leaves, and slightly tabular in orientation, despite still preserving the highly porous, chaotic arrangement (Fig. 3C).

### Carbonate facies in the fossil tufa

#### Boundstones of stromatolites

This facies appears along outcrop 1 as domical stromatolites preserved in tabular beds and intercalated with phytoclastic rudstones. The dome-shaped nodules consist of bulges 6–10 cm wide (Fig. 2C), composed of cyclical isopachous fringes of calcite spar and micrite, deposited centripetally around shrubs and stems (Fig. 2D, E). Each of these fringes ranges on a micrometric scale.

#### Rudstones of phytoclasts

This facies comprises tabular, non-organized, laterally continuous beds ranging in thickness from 40 to 300 cm. Coated stems (0.5 to 25 cm wide) are very common. Interior cavities of stem molds are empty (Fig. 2F, G, I; Fig. 3D-H). The coating comprises radiating sparry shrubs (Fig. 2G; Fig. 3E-F), and the calcitic cement, including micrite, pseudosparite, microsparite, and sparite. In outcrop 2, specifically, the contact between spar and micrite is preserved in both graded (Fig. 3J, K) and non-graded (Fig. 3L, M) zones, with pseudosparite being more abundant in graded zones (Fig. 3J, K). The microsparitic texture occurs in association with sparry and cloudy calcites (Fig. 3G, H). Both spar and microsparite show bladed, dog-tooth-like morphology (Fig. 3G, H). Some stem molds exhibit inner walls composed of microsparitic microbial filaments (Fig. 2I). Leaf molds are abundant, preserved with empty interior cavities in outcrop 1 (Fig. 2.F, H, I), and their surface often presents either radial shrubs (Fig. 2H). Some leaf molds show fringes of dog-tooth-like to bladed spar crystals, presenting equidimensional, cloudy calcite with irregular contacts associated with dark beige micrite (pseudosparite; Fig. 2I). Furthermore, leaf countercasts from outcrop 2 show a complex interplay of micrite, spar, microspar, pseudospar, and shrubs (Fig. 3J-M). The thick fringes that form these countercasts consist of bladed calcite (Fig. 3E). In these areas, shrubs occur superimposed on isopachous lines of micrite and pseudosparite (Fig. 3E, J, M). Gastropod molds are occasionally found among the macrophytes, but not in sufficient abundance to warrant a distinct facies classification (Fig. S1).

### Cathodoluminescence

Crystals show dull to almost no luminescence (Fig. S2). Colors range from yellow to orange and reddish brown. Luminescence is somewhat homogenous, with spar and microspar appearing brighter (yellowish and orange) than micrite, which exhibits dull, reddish-brown tones.

### Scanning electron microscopy (SEM) and EDS

**Fig. 4:**
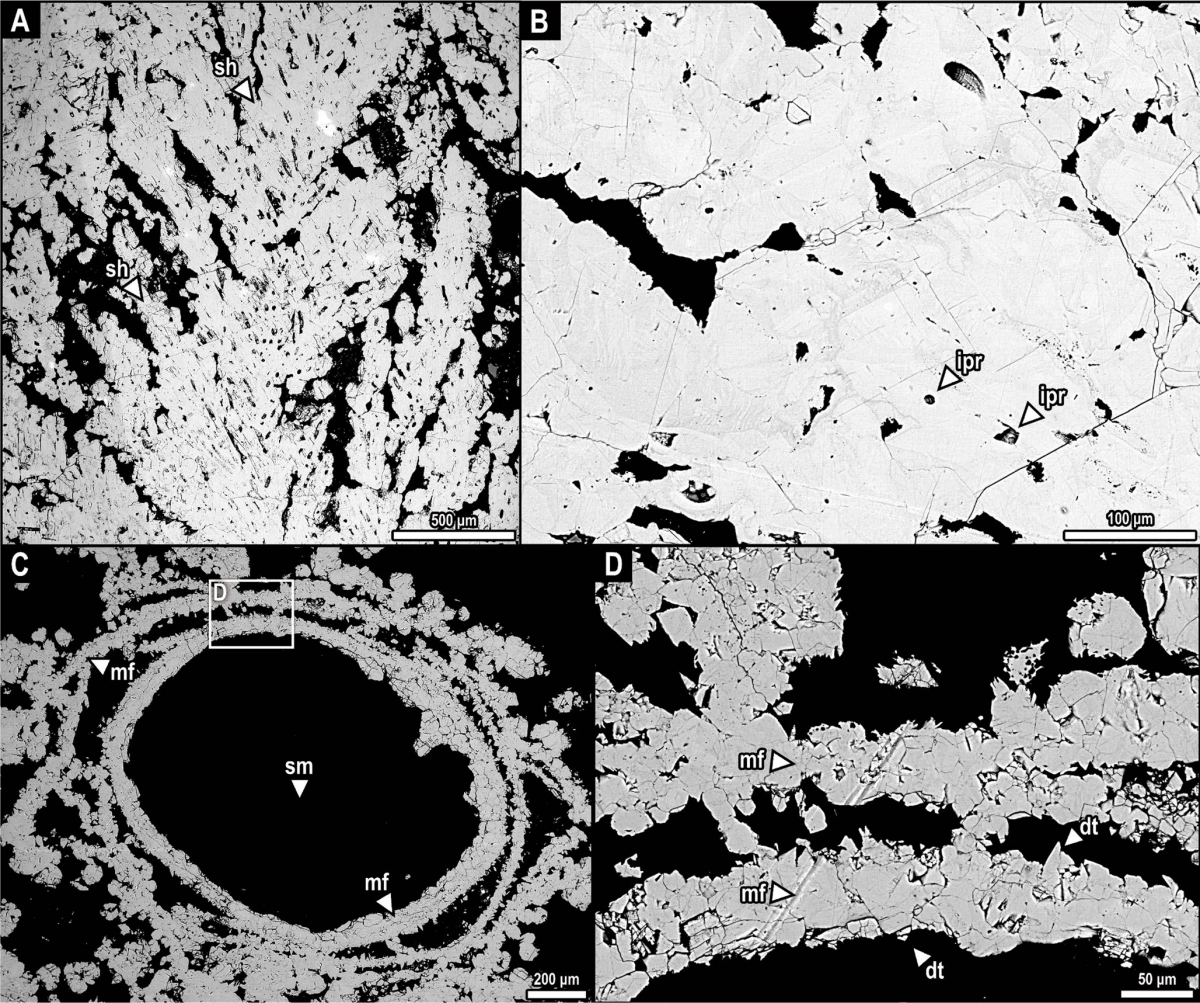
Scanning Electron Microscopy (SEM) images of phytoclastic rudstones from the Quixeré site. A, shrubs developed around the stem moldic porosity. B, intracrystalline porosity in calcite cementum. C, microbial filaments preserved as dog-tooth-like, bladed, radially developed cement around a stem mold. D, detail of the spar and microspar crystals from the microbial filaments. **Abbreviations**: dt, dog-tooth-like to bladed spar calcite; ipr, intracrystalline porosity; mf, microbial filament; sh, shrub; sm, stem mold

High-resolution SEM imagery (Fig. 4) supports these findings, providing a clear visualization of radial shrub networks (Fig. 4.A) and intracrystalline porosity in calcite cement (Fig. 4.B). Stem molds are surrounded by interspersed relic microbial filaments and biofilm residuals (Fig. 4.C), preserved as dogtooth-like, bladed calcite cementum (Fig. 4.D). The minerals identified by EDS are predominantly low-Mg calcite (Fig. S3,S4). Detrital quartz and clays are nearly absent.

### Taphonomy

**Fig. 5:**
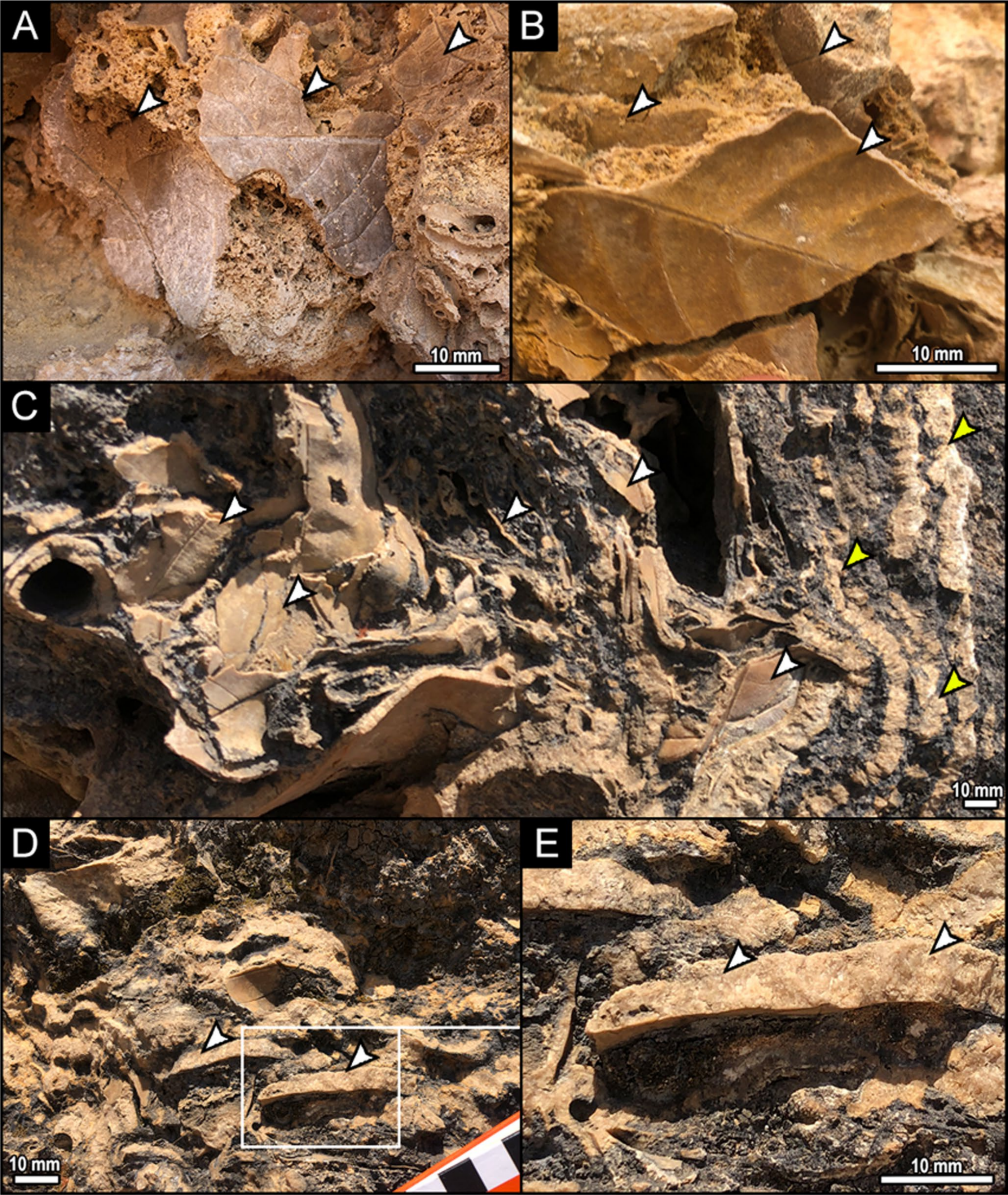
Fossil macrophytes in Quaternary tufas from the Quixeré site. A, B, accumulations of angiosperm leaf countercasts (arrows). C, association of angiosperm leaf countercasts (white arrow) encapsulated by crustal shrubs (yellow arrows). D, three-dimensional leaves in a horizontal position in profile view, with mineral precipitation forming countercasts (arrows). E, detail of D

The phytoclastic rudstone beds of this deposit consist of dense layers of leaf imprints, some arranged horizontally and others chaotically, with no preferred orientation (Fig. 5). Leaf sizes range from 50 to 100 mm in length.

The fossil assemblage of the Quixeré/CE tufa is essentially parautochthonous, with layers of horizontal leaves formed by gravitational deposition or slow flow action in underwater environments, such as pools or natural cavities. In contrast, the chaotic packages, without preferential orientation, were formed by turbulent flows or by the trapping of plant matter by algal filaments in shallow pools or dam areas.

In general, the preservation of the phytofossils includes evidence of secondary precipitation of sparry cements that filled the empty spaces left after the degradation of organic matter. This phenomenon resulted in the formation of countercasts of leaves and branches (Fig. 5.D-E), significantly reducing the macroscopic porosity of the rock. In addition, some leaf bodies show apparent transverse dilation, possibly caused by secondary growth of minerals within the casts (Fig. 5.C-E).

### Catalog of fossil flora morphotypes

**Fig. 6:**
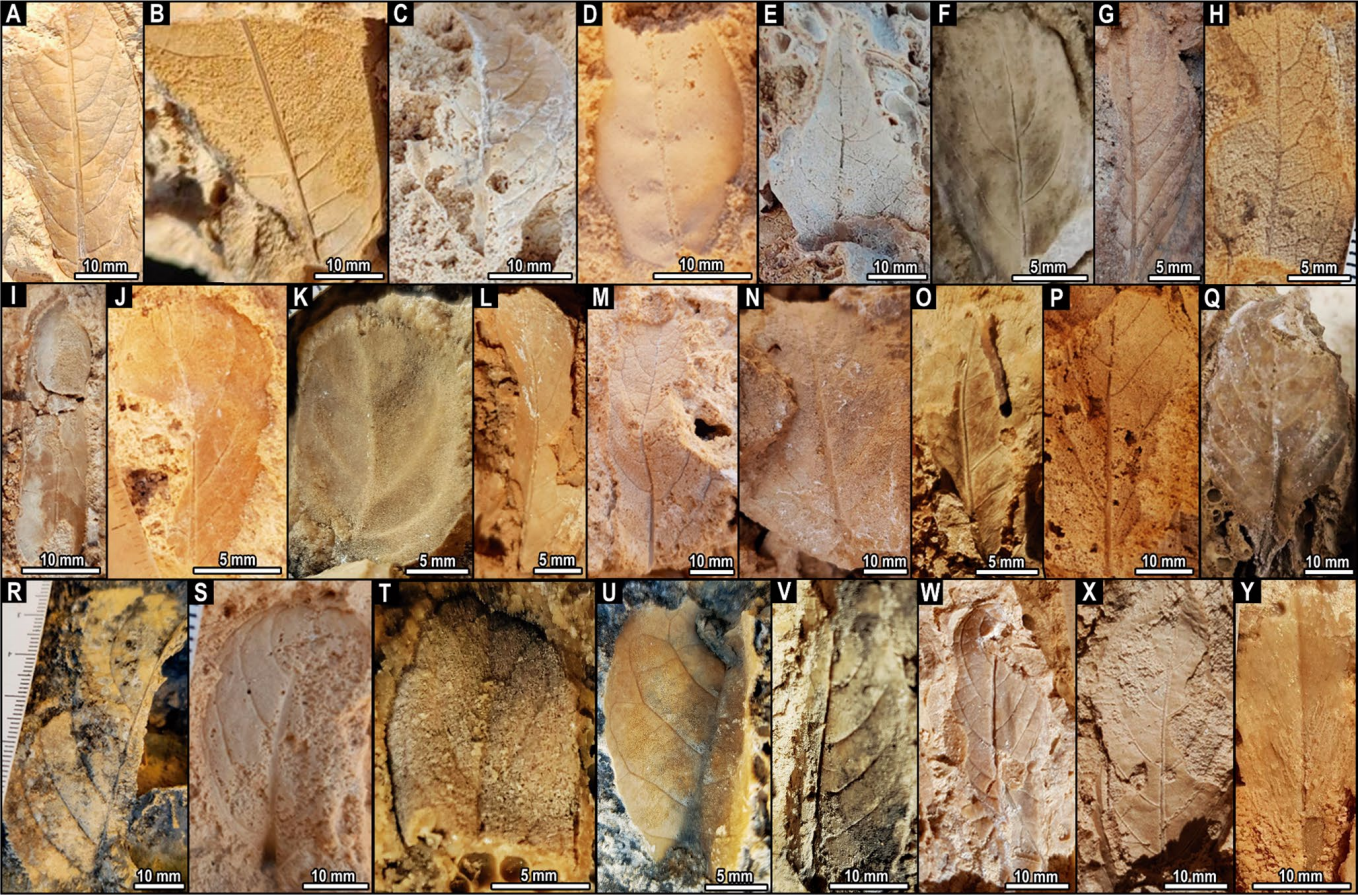
Leaf morphotypes preserved as molds, imprints, and countercasts in the Quaternary tufas of the Quixeré site. **A**, morphotype MQ-1. **B**, MQ-2. **C**, MQ-3. **D**, MQ-4. **E**, MQ-5. **F**, MQ-6. **G**, MQ-7. **H**, MQ-8. **I**, MQ-9. **J**, MQ-10. **K**, MQ-11. **L**, MQ-12. **M**, MQ-13. **N**, MQ-14. **O**, MQ-15. **P**, MQ-16. **Q**, MQ-17. **R**, MQ-18. **S**, MQ-19. **T**, MQ-20. **U**, MQ-21. **V**, MQ-22. **W**, MQ-23. **X**, MQ-24. **Y**, MQ-25. Photographs adjusted for contrast and brightness

Fossil leaf impressions of angiosperms are abundant, with thirty-six specimens yielding twenty-five morphotypes classified by margin, venation, and lamina size (Fig. 6; Table 1). Most are microphylls and nanophylls, with a single notophyll, displaying entire or subtly dentate margins and a spectrum of elliptical to lanceolate geometries.

**Table 1:** Properties of leaf morphotypes preserved as molds, imprints, and countercasts in the Quaternary tufas of the Quixeré site. All morphotypes present one basal vein

Morphotype	Width (mm)	Length (mm)	Size class	Blade shape	Margin	Base	Apex	Primary venation	Secondary venation	Tertiary venation
MQ-1	16	47	Microphyll	Elliptic	Straight	Acute	-	Pinnate	Alternate, brochidodromous pattern	Sinuous with obtuse angle to midvein
MQ-2	34	35	Microphyll	Elliptic	Straight	-	-	Pinnate	Alternate, brochidodromous pattern	Straight with obtuse angle
MQ-3	15	40	Microphyll	Elliptic	Straight	Acute	-	Pinnate	Alternate, brochidodromous pattern	Straight with obtuse angle
MQ-4	17	26	Nanophyll, Microphyll, Notophyll	Elliptic	Straight	Acute	-	Pinnate	Symmetrical, brachidromous pattern	-
MQ-5	26	40	Not used	-	-	-	-	Pinnate	Alternate, brochidodromous pattern	Sinuous with obtuse angle
MQ-6	11.6	20	Nanophyll	Elliptic	Straight	-	-	Pinnate	Alternate, brochidodromous pattern	-
MQ-7	20	46	Microphyll	Lanceolate	Straight	Acute	Straight	Sub-alternate	Alternate, brochidodromous pattern	Straight with obtuse angle
MQ-8	10	20	Nanophyll	Elliptic	-	-	-	Pinnate	Alternate, semi-craspedodromous pattern	Obtuse with chevron pattern
MQ-9	9	38	Nanophyll	Linear	Straight	-	Convex	Pinnate	Alternate, craspedodromous pattern	Not apparent
MQ-10	18	31	Nanophyll	Obovate	Straight	-	Convex	Pinnate	Symmetrical, brochidodromous pattern	Straight and obtuse
MQ-11	14	19	Nanophyll	Elliptic	Straight	-	Convex	Pinnate	Parallel, brochidodromous pattern	Not apparent
MQ-12	13	36	Nanophyll	Lanceolate	-	-	-	One primary vein	Alternate, brochidodromous pattern	-
MQ-13	50	80	Microphyll	Notophyllous	-	-	-	Pinnate	Alternate, brochidodromous pattern	Straight with obtuse angle
MQ-14	50	70	Nanophyll	Elliptic	Straight	-	-	Pinnate	Opposite, brochidodromous pattern	-
MQ-15	17	23	Nanophyll	Elliptic	Straight	Obtuse	-	Pinnate	Opposite	Sinuous and obtuse
MQ-16	29	49	Microphyll	Elliptic	Straight	-	-	Pinnate	Alternate	Sinuous, obtuse
MQ-17	33	76	Microphyll	Elliptic	Straight	-	-	Pinnate	Alternate, brochidodromous pattern	Straight with obtuse angle
MQ-18	48	73	Microphyll	-	Straight	-	-	Pinnate	Brochidodromous pattern	Straight with obtuse angle
MQ-19	15	25	Nanophyll	Elliptic	Straight	Acute	-	Pinnate	Alternate, brochidodromous pattern	Straight with obtuse angle
MQ-20	11	18	Nanophyll	-	Crenate	-	-	Pinnate	Alternate, brochidodromous pattern	Sinuous
MQ-21	40	48	Microphyll	-	Straight	-	-	Pinnate	Alternate, brochidodromous pattern	Straight with obtuse angle
MQ-22	32	52	Microphyll	Lanceolate	Straight	-	-	Pinnate	Alternate, brochidodromous pattern	Straight with obtuse angle
MQ-23	21	60	Microphyll	Lanceolate	Straight	-	-	One primary vein	Alternate, brochidodromous pattern	Straight with obtuse angle
MQ-24	30	60	Microphyll	Elliptic	Straight	-	-	Pinnate	Alternate	-
MQ-25	12	19	Nanophyll	Elliptic	Straight	-	-	Pinnate	Alternate	-

### Paleoclimatic reconstruction

The measured anatomical data from these leaves provide sufficient preservation for paleoclimatic inferences. Estimates of mean annual temperature (MAT), derived via leaf margin analysis, consistently fall between 25.4 °C and 25.9 °C (see Tables S2-S4). In contrast, leaf area calculations project mean annual precipitation (MAP) ranges from 515 to 779 mm/year (Table S3), altogether attesting to a humid tropical paleoenvironment in the Quaternary (Hagen 2019; Santiago and Ricardi-Branco 2020; Toumoulin et al. 2020). 

### Quantitative porosity analysis

Quantitative assessment of porosity involves image segmentation and digital analysis of blue-dye-impregnated thin sections (see the supplementary material). Moldic porosity, resulting from the dissolution and packing of organic matter, is prominent and varies from approximately 34% to approximately 60% (see Table S5), depending on microfacies and mold concentration. Intracrystalline pores are restricted to zones of intense shrub and microbial crystal development, but they contribute a locally significant pore volume. Microbial mat zones, particularly those characterized by fenestral or domal laminations, display interconnected porosity networks that extend throughout the carbonate cement. Image-derived porosity calculations align with macroscopic and point-count estimates, confirming the relationship between primary biological input, early microbial cementation, and the final pore network architecture (Vidal et al. 2019). The results collectively reveal a depositional system fundamentally shaped by structural controls, episodic organic influx, and microbial precipitation, resulting in tufa bodies exhibiting wide heterogeneity in both texture and pore architecture.

## Discussion

The sampled tufa bodies in Quixeré exhibit remarkable lithological diversity, with dense accumulations of phytoclastic material, including well-preserved leaf and stem molds, and leaf countercasts, embedded chaotically within micritic and sparitic cements. The observed facies correspond to phytoclastic rudstones and stromatolitic boundstones (Arenas-Abad et al. 2010), characterized by a highly irregular packing of phytoclasts and variable fabric textures, which collectively point to fluctuating depositional energy and the episodic influx of fresh organic debris in a karst-controlled, hydrologically dynamic system (Barbosa 2013; Maia and Bezerra 2014; Oste et al. 2021, 2023).

### Lithification, diagenesis, and karstification

Lithification represents the first modification in the formation of a tufa deposit, driven by microbial influence on carbonate precipitation, seasonal desiccation, and calcite supersaturation within micropores governed by capillarity (Dupraz et al. 2009; García-del-Cura et al. 2012). In the context of tufa diagenesis, neomorphism refers to the in-situ recrystallization of primary carbonate minerals through a process of dissolution and reprecipitation (Flügel 2010; Folk 1965). It manifests as a textural coarsening where finer, primary micrite crystals (< 4 μm) are progressively replaced by larger crystals of microspar (5–30 μm) and eventually pseudospar, a process known as aggrading neomorphism (Armenteros 2010; Love and Chafetz 1988; Oste et al. 2023). Unlike cementation, which fills pore space, neomorphism primarily redistributes calcite, resulting in no significant change in total porosity (Armenteros 2010). In tufa systems composed of stable low-magnesium calcite, the driving force for this recrystallization is not mineralogical instability but rather the high surface energy of the initial fine-grained micrite, which favors the growth of larger, more thermodynamically stable crystals over time (Oste et al. 2023).

The tufas at the Quixeré site exhibit the rapid induration characteristic of continental carbonates, where early diagenesis occurs concurrently with textural coarsening, resulting in minimal porosity loss (Oste et al. 2023). Primary micrite precipitated around and within microbial filaments, with aggrading neomorphism to microspar and locally to pseudospar via dissolution-precipitation (Armenteros 2010; Dupraz et al. 2009; Erik Flügel 2009; Folk 1965). Despite the stable low-magnesium calcite, crystal-size instability and the high surface area of lime mud drive recrystallization (Oste et al. 2023). The neomorphic pathway preserves pore volume, consistent with similar pore percentages in modern and fossil samples (Vidal et al. 2019). The grading from micrite to microspar in both sections corroborates the interpretation of aggrading neomorphism as a principal pathway of early diagenesis (Erik Flügel 2009; Folk 1965; Oste et al. 2021, 2023). However, the enhanced spar and pseudospar content in outcrop 2 reflects a more extensive calcite coarsening, crystal rearrangement, and cementation (Oste et al. 2023). This progression is typical of subaerial exposure or shallow burial settings, where early diagenesis involves dissolution-precipitation cycles driven by changing saturation indices and microbial mediation, as observed in continental tufa systems (Dupraz et al. 2009; García-del-Cura et al. 2012; Oste et al. 2021, 2023). Furthermore, our cathodoluminescence data suggest a well-oxygenated environment, with a low Mn²**⁺**/Fe²**⁺** ratio, indicating an oxidizing meteoric paleoenvironment (Machel 2000; Scholle and Ulmer-Scholle 2003; Shtober-Zisu et al. 2020).

The petrography of outcrop 2 samples reveals an abundance of leaf countercasts and a complex interplay of micrite, spar, microsparite, pseudospar, and shrub calcite, providing important insights into the progression of diagenetic maturation compared to Outcrop 1. Outcrop 2 displays more advanced neomorphic textures, with a greater presence of pseudospar and an intricate association of multiple crystalline phases within the countercast rims. These features contrast with the simpler micritic and spar fabrics dominant in outcrop 1, indicating that outcrop 2 represents a later stage in diagenetic evolution (Oste et al. 2021, 2023). The presence of leaf countercasts in outcrop 2, which are mineral infillings of voids left by decayed leaves, demonstrates extended interaction with diagenetic fluids that drove advanced neomorphic recrystallization processes (Oste et al. 2021, 2023). The thick fringes of bladed calcite crystals and superimposed shrubs observed on the countercast surfaces imply recurrent episodes of supersaturation and crystal growth, characteristic of evolving pore water chemistry during burial or exposure in vadose zones (Armenteros 2010; Capezzuoli et al. 2014; Oste et al. 2021; Martyn Pedley 2014).

Differences in karstification are also evident between the two outcrops. Outcrop 1, with its prominent empty leaf molds and pervasive microbial fabrics, retains high primary porosity and marks the initial phase of structural collapse-related karst development, as evidenced by the presence of large cavities. Conversely, outcrop 2 exhibits partial infilling of moldic pores by spar cements (forming the countercasts), indicating a progression toward porosity occlusion as diagenesis and karstification advance (Maia and Bezerra 2014; Oste et al. 2023). The interplay of enhanced karst dissolution, collapse features, and subsequent cementation in outcrop 2 exemplifies a later stage of maturation within the same karst-controlled sedimentary basin.

**Fig. 7:**
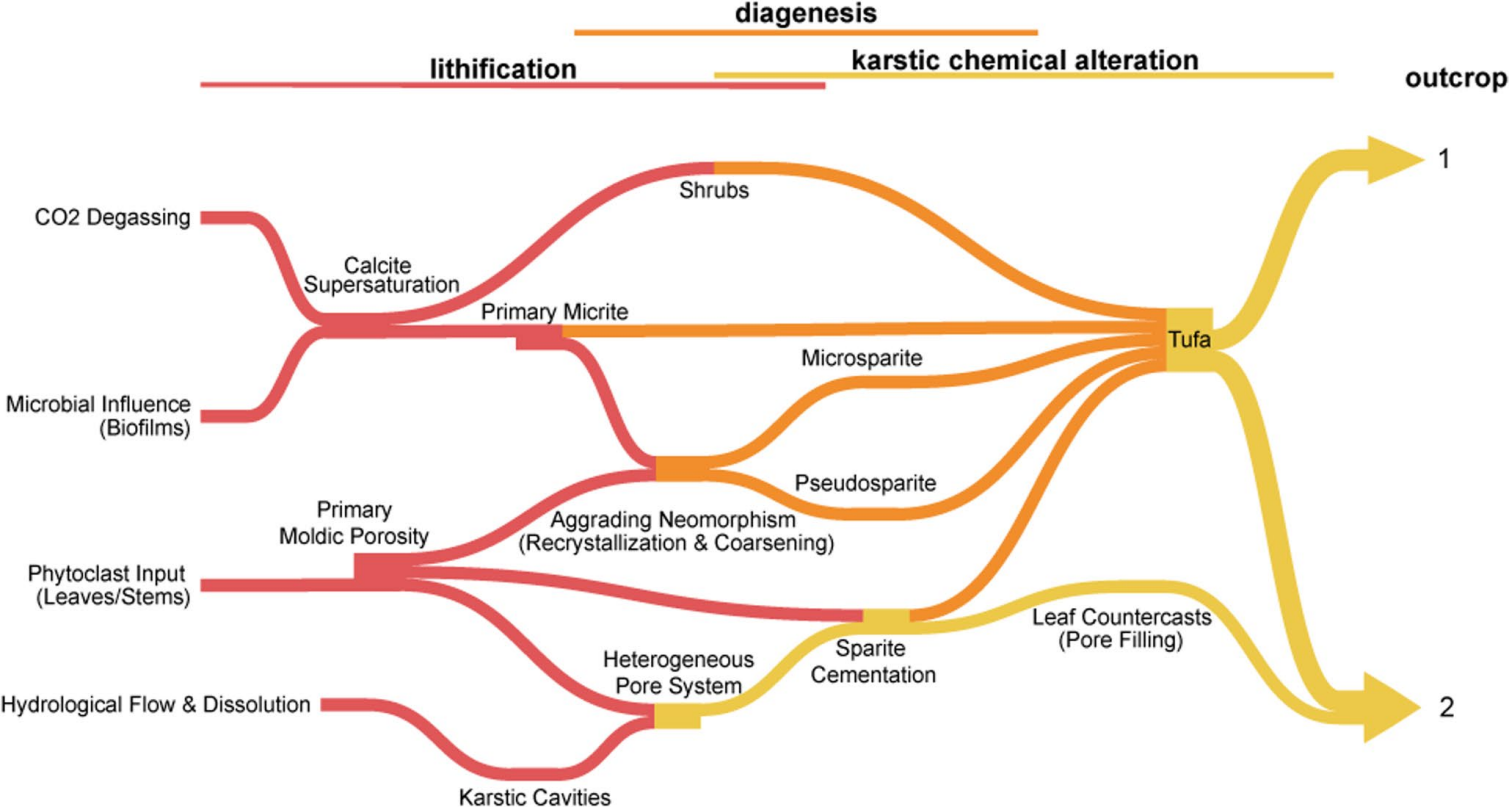
Flow diagram model of tufa lithification, diagenesis, and karstification for the Quixeré site (outcrops 1 and 2)

**Fig. 8:**
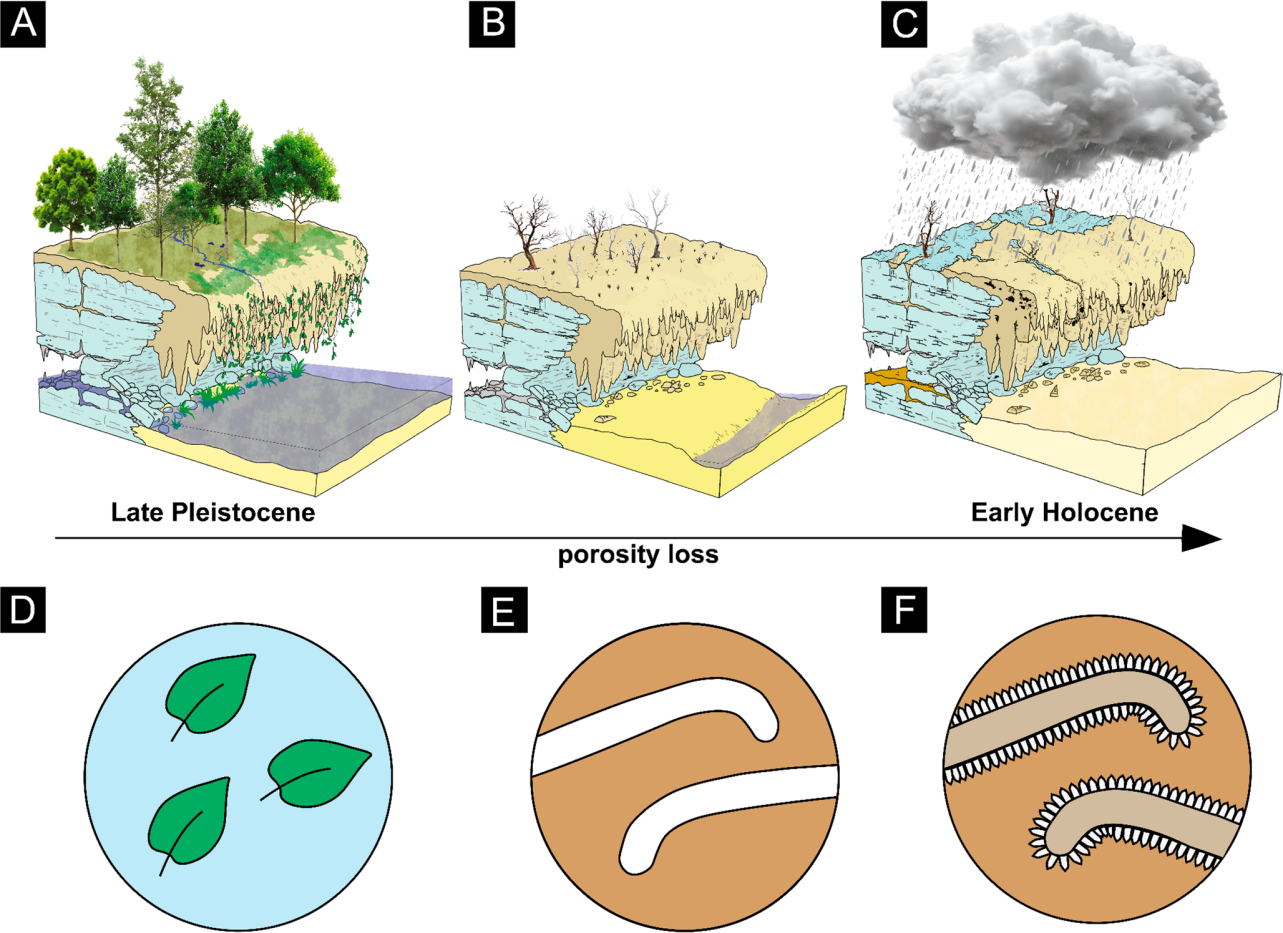
Hypothetical evolutionary model for the environment, vegetation and tufa formation in the western Potiguar Basin during the Quaternary at the Quixeré site, Ceará. Schematic models of the site in A-C, and the process of fossil diagenesis and karstification at the sample level in D-F. A, active tufas amidst a tropical cerrado-like forest during the Pleistocene, and accumulations of phytoclasts in pools (D). B, increasing aridity and xeromorphic adaptation of the vegetation during the Early Holocene. Phytoclasts decomposed, generating moldic porosity (E). C, increasing aridity with rare seasonal rainy seasons, xeromorphic caatinga, and karstification is widespread, resulting in the opening of cavities and localized cave collapses in parts of the outcrop, during the Late Holocene. F, dissolved calcite precipitates in the molds, generating countercasts coated with ‘dog-tooth’ sparite and decreasing porosity at the microscopic scale

Therefore, the petrographic and taphonomic evidence delineates a clear diagenetic progression (Fig. 7) from the microbially-dominated, high-porosity fabrics of outcrop 1 (Fig. 8.A, B) to the more recrystallized, cement-rich, and texturally mature carbonates of outcrop 2 (Fig. 8.C), reflecting prolonged diagenetic alteration within the same karstic system.

### Climate change and the evolution of vegetation

The Quaternary tufa deposits at Quixeré serve as a high-resolution archive of the significant climatic and ecological transformations that occurred in the area where the Caatinga is located today. The paleobotanical data from this study provide direct evidence of a past humid tropical climate during the Pleistocene-Holocene transition, starkly contrasting with the region’s modern semi-arid conditions. Paleoclimatic reconstructions, based on the twenty-five mesophytic leaf morphotypes dominated by microphylls and nanophylls, quantify this past environment with a mean annual temperature (MAT) of 25.4–25.9 °C and a mean annual precipitation (MAP) of 515–779 mm/year. This lush, structurally complex vegetation, rich in angiosperms, flourished during wetter periods of the Quaternary, which are recognized regionally as having a more humid and warmer climate than today (Assine and Soares 2004; Sallun Filho and Karmann 2007). The excellent preservation of this diverse flora within the tufa is itself an indicator of a productive environment with abundant organic matter input from gallery forests lining the fluvial system (Ford and Pedley 1996).

The transition from this past humid ecosystem to the modern xeromorphic-adapted Caatinga biome represents a major paleoecological shift. This climatic drying trend is recorded in the geological succession by the sharp, erosional contact observed between Pleistocene and Holocene tufa units in other localities in Brazil, marking periods where tufa deposition ceased and fluvial incision dominated (E. C. Oliveira et al. 2017). The modern Caatinga is defined by its physiological and anatomical adaptations to chronic water deficits, including reduced leaf area and high sclerophylly (Albuquerque et al. 2012; Fernandes et al. 2022), traits that stand in direct opposition to the large, entire-margined leaves preserved in the Quixeré tufas (Fernandes et al. 2022). The waning of tufa-forming conditions is a well-documented phenomenon that often coincides with late-Holocene climate shifts toward increased aridity (Andrews 2006; Goudie et al. 1993).

The interplay between vegetation and diagenesis further records this environmental evolution. The high primary moldic porosity (over 30%) observed in the phytoclastic rudstones is a direct consequence of the rapid burial and subsequent decay of abundant plant material from the ancient mesophytic forest (Ford and Pedley 1996). This process demonstrates that the system was sufficiently productive to supply a high volume of organic matter (Arenas-Abad et al. 2010; Gradziński 2010). As the climate became progressively drier, the decline in vegetation cover and reduced water flow would have not only halted tufa accretion but also altered the diagenetic regime, promoting subaerial exposure and enhancing karstification processes that further modified the landscape (Maia and Bezerra 2014; Oste et al. 2021). The Quixeré deposits, therefore, do not just capture a static snapshot of a past climate but preserve the physical and biological evidence of a dynamic environmental turnover from a humid, forested landscape to a semi-arid, xeromorphic ecosystem.

## Conclusions

The Quaternary tufa deposits of Quixeré, Brazil, studied through an integrated sedimentological, petrographic, and paleobotanical approach, chronicle a dynamic history of environmental change in the Potiguar Basin. The study reveals a clear diagenetic progression, advancing from high primary porosity and microbially-dominated fabrics to more texturally mature carbonates, characterized by advanced neomorphism and spar-cemented countercasts. This maturation gradient, occurring within a landscape structurally controlled by karst-related collapse features, records the long-term diagenetic response to fluctuating vadose and phreatic conditions. Furthermore, paleobotanical analysis quantifies a major climatic turnover, from a humid tropical environment that supported mesophytic forests to the semi-arid, xeromorphic-adapted Caatinga biome of today. These tufas, therefore, are not merely static indicators of a past climate but are high-resolution archives that document the coupled evolution of diagenesis, karst processes, and vegetation in response to Quaternary climate change, reinforcing their value as predictive analogues for both tropical paleoecology and heterogeneous carbonate reservoirs.

## Data Availability

Python is an open-source programming language. The download link for the software and its packages used in this study is provided in the Materials and Methods section. All data used in the analyses are provided in the supporting information and have been deposited in the Morphobank repository and can be accessed through the link: https://www.morphobank.org/permalink/?P6161.

## Declarations

### Open research

Python is an open-source programming language. The download link for the software and its packages used in this study is provided in the Materials and Methods section. All data used in the analysis are available as supporting information. All data used in the analyses are provided in the supporting information and have been deposited in the Morphobank repository and can be accessed through the link: https://www.morphobank.org/permalink/?P6161.
